# Supplementary material for: Health professionals practice and associated factors towards precautionary measures for COVID-19 pandemic in public health facilities of Gamo zone, southern Ethiopia: A cross-sectional study
Source: PLoS One. 2021 Mar 9;16(3):e0248272. doi: 10.1371/journal.pone.0248272 (PMC7943004; doi:10.1371/journal.pone.0248272)
Supplement: S1 File — (PDF) [file pone.0248272.s001.pdf]

English Version Survey Tool

ARBA MINCH UNIVERSITY

COLLEGE OF MEDICINE AND HEALTH SCIENCES

Dear participants

This questionnaire is prepared to assess the health professionals' practice and associated factors towards precautionary measures for COVID-19 pandemic in public health facilities of the Gamo zone, southern Ethiopia, 2020. The questionnaire contains both closed and open-ended questions and you will respond based on the questions. You therefore kindly requested to provide a genuine response to the questions. The information you provided is confidential and used only for this study. If you have any questions, don‘t hesitate to ask the data collector.

Your cooperation and participation until the completion of the response are very necessary for the successful completion of the assessment.

Thank you in advance for your cooperation!!!

Data collectors sign: _____________

Identification Number [ ]

Questionnaire developed to assess the health professionals practice and associated factors towards precautionary measures for COVID-19 pandemic in public health facilities of Gamo zone, southern Ethiopia, 2020

**SNo**	**Questions**	**Response**	**Skip**
**Part I: Socio-demographic and professional-related characteristics**			
**1.**	Sex	a. Male<br>b. Female	
**2.**	Age	_________(in year)	
**3.**	What is the educational level?	a. Diploma<br>b. BSc<br>c. MSc<br>d. GP<br>e. Specialist	
**4.**	What is your profession or job category?	__________________________	
**5.**	Work experience	____________(in complete year)	
**6.**	Working hours per day in the institution	__________(in hour)	
**7.**	What source that you get information regarding the COVID-19 pandemic?<br><br>*(Multiple responses if possible)*	a. Seniors & Other Colleagues<br>b. Newspapers & Magazines<br>c. Posters & Pamphlets<br>d. Seminars & workshops<br>e. Radio & television<br>f. Social Media<br>g. Other (________________)	

**Part II: Knowledge related question regarding COVID-19**				
**SNo**	**Characteristics**	**Yes**	**No**	**Remark**
**1.**	The influenza vaccine also gives protection from COVID-19.			
**2.**	Special caution must take if a person presents fever, cough, and sneezing.			
**3.**	The main source of the virus may be Plant.			
**4.**	COVID-19 patients develop severe acute respiratory illness			
**5.**	Washing hands vigorously (soap/water(can prevent COVID-19			
**6.**	COVID-19 spreads through close contact like caring and/or shaking			
**7.**	People with comorbidity like diabetes and hypertension risk of death with COVID-19			
**8.**	Polymerase chain reaction (PCR) can be used to test COVID-19			

**9.**	Vaccination of coronavirus disease is available			
**10.**	Fever, cough, and shortness of breath are symptoms for COVID-19			
**11.**	Antibiotics are first-line treatment			
**12.**	The incubation period for the virus is 2-14 days			
**13.**	Coronavirus infection could be fatal			
**14.**	COVID-19 is a viral infection			

**Part III: Attitude related question towards precautionary measures for COVID-19 pandemic: 5 points Likert scale**							
**SNo**	**Characteristics**	**1**	**2**	**3**	**4**	**5**	**Remark**
**1.**	Healthcare workers must acknowledge themselves with all the information about COVID-19						
**2.**	Transmission of COVID-19 infection can be prevented by using universal precautions given by WHO, CDC						
**3.**	Any related information about COVID-19 should be disseminated among healthcare workers						
**4.**	Prevalence of COVID-19 can be reduced by the active participation of healthcare workers in the health facility infection control program						
**5.**	Intensive and Emergency treatment should be given to diagnosed patients.						
**6.**	COVID-19 patients should be kept in isolation						
**7.**	Gowns, gloves, masks, and goggles must use when dealing with COVID-19 patients?						

Remark: strongly disagree (1), disagree (2), neutral (3), agree (4), and strongly agree (5)

**Part IV: Practice related questions regarding precautionary measures for COVID-19**					
**SNo**	**Characteristics**	**1**	**2**	**3**	**Remark**
**1.**	Do you educate your patient about the disease?				
**2.**	Do you use a facemask in crowds?				
**3.**	Do you avoid touching your eyes, nose, or mouth as far as you can?				
**4.**	Do you throw the used tissue in the trash?				
**5.**	Do you cover your nose and mouth with a tissue during sneezing or coughing?				
**6.**	Do you use soap or hand sanitizer to wash your hands continuously?				

Remark: No (1), Sometimes (2), and Yes (3)

Observational Checklist

SNo	Points to be considered	1	2	3
**1.**	Hand hygiene stations (water, soap, paper towel, alcohol-hand rub), and waste bins are installed at strategic locations across the health facility.			
**2.**	Health care workers applying standard precautions for all patients.			
**3.**	Droplets and contact precautions are recommended			
**4.**	Patients placed in the adequately ventilated rooms			
**5.**	A one-meter distance between beds maintained			
**6.**	Equipment is either single-use or disposable or if equipment (e.g., stethoscopes, blood pressure cuffs, thermometers, food trays) needs to be shared among patients, clean and disinfect between use for each patient (e.g., by using ethyl alcohol 70%)			
**7.**	Routinely clean and disinfect surfaces with which the patient is in contact			
**8.**	Health care worker are applying droplet and contact precautions before entering the room			
**9.**	Health care workers are applying airborne precautions for aerosol-generating procedures, such as tracheal intubation, non-invasive ventilation, tracheotomy, cardiopulmonary resuscitation, manual ventilation before intubation, bronchoscopy, collection of nasopharyngeal swap/aspirate and autopsy.			
**10.**	Team of HCWs should bed signaled to care exclusively for suspected or confirmed cases to reduce the risk of transmission.			
**11.**	Staff (HCW, cleaning personnel) receive training on standard, contact, droplets, and airborne precautions (including correct use of PPE, donning and doffing, masks tested for fitting, hand hygiene, respiratory hygiene, etc.).			
**12.**	Adequate personal protective equipment (PPE) (i.e., medical/surgical masks, N95/FFP2 respirators, gloves, gowns, eye protection) is easily accessible to staff. If the supply of PPE is limited, prioritize staff caring for cases.			
**13.**	Avoid moving and transporting patients out of their room or area unless medically necessary.			
**14.**	HCWs who are transporting patients perform hand hygiene and wear appropriate PPE.			
**15.**	The area receiving the patient arrange for all necessary precautions as early as possible before the patient's arrival			
**16.**	Visitors those essential for patient support are limited			
**17.**	Visitors apply droplet and contact precautions.			
**18.**	All persons entering the patient's room, including all staff and visitors recorded.			
**19.**	Manage laboratory specimens, laundry, food service utensils, and medical waste following safe routine procedures according to IPC guidelines.			

Remark: not practiced (1), sometimes (2), and always practiced (3)
